# Positive and negative ions of the amino acid histidine formed in low‐energy electron collisions

**DOI:** 10.1002/jms.4427

**Published:** 2019-11-06

**Authors:** Rebecca Meißner, Linda Feketeová, Andreas Bayer, Johannes Postler, Paulo Limão‐Vieira, Stephan Denifl

**Affiliations:** ^1^ Institut für Ionenphysik und Angewandte Physik and Center for Molecular Biosciences Innsbruck (CMBI) Universität Innsbruck Technikerstraße 25 6020 Innsbruck Austria; ^2^ Atomic and Molecular Collisions Laboratory, CEFITEC, Department of Physics Universidade NOVA de Lisboa 2829‐516 Caparica Portugal; ^3^ Institut de Physique Nucléaire de Lyon; CNRS/IN2P3, UMR5822 Université de Lyon, Université Claude Bernard Lyon 1 43 Bd du 11 novembre 1918 69622 Villeurbanne France

**Keywords:** amino acids, anions, cleavage reactions, ionisation potentials, mass spectrometry

## Abstract

Histidine is an aromatic amino acid crucial for the biological functioning of proteins and enzymes. When biological matter is exposed to ionising radiation, highly energetic particles interact with the surrounding tissue which leads to efficient formation of low‐energy electrons. In the present study, the interaction of low‐energy electrons with gas‐phase histidine is studied at a molecular level in order to extend the knowledge of electron‐induced reactions with amino acids. We report both on the formation of positive ions formed by electron ionisation and negative ions induced by electron attachment. The experimental data were complemented by quantum chemical calculations. Specifically, the free energies for possible fragmentation reactions were derived for the τ and the π tautomer of histidine to get insight into the structures of the formed ions and the corresponding neutrals. We report the experimental ionisation energy of (8.48 ± 0.03) eV for histidine which is in good agreement with the calculated vertical ionisation energy. In the case of negative ions, the dehydrogenated parent anion is the anion with the highest mass observed upon dissociative electron attachment. The comparison of experimental and computational results was also performed in view of a possible thermal decomposition of histidine during the experiments, since the sample was sublimated in the experiment by resistive heating of an oven. Overall, the present study demonstrates the effects of electrons as secondary particles in the chemical degradation of histidine. The reactions induced by those electrons differ when comparing positive and negative ion formation. While for negative ions, simple bond cleav ages prevail, the observed fragment cations exhibit partly restructuring of the molecule during the dissociation process.

## 1 INTRODUCTION

The ever‐increasing amount of ionising radiation to which human beings are exposed to in modern society is understood to be the major cause of damage to living cells.^1^, ^2^ Electrons are one of the most abundant secondary species formed after primary radiation impact with yields of around 5 × 10^4^ per MeV deposited energy of the incident radiation^3^, ^4^ and kinetic energies < 20 eV. Also in the case of ion beam tools for cancer therapy, it was shown that a large majority of secondary electrons produced have kinetic energies < 30 eV.^5^, ^6^ These electrons can react with molecular components of the cell to form positive and negative ions and radicals and can substantially contribute to the cell damage through sugar modifications, base release, and single strand and double strand breaks.^7^, ^8^

Thus, the underlying processes of the damage and the repair of biologically essential molecules, such as DNA and proteins, are of great importance and a subject of intense research. Especially the onset of the different processes induced by low‐energy electrons leading to formation of positive and negative ions and radicals is crucial to provide more accurate predictions for ion beam cancer therapy, shielding of human space missions, prediction of the consequences of exposure to radiation, and for possible formation and detection of amino acids in extraterrestrial environments.^9^

**Scheme 1:**
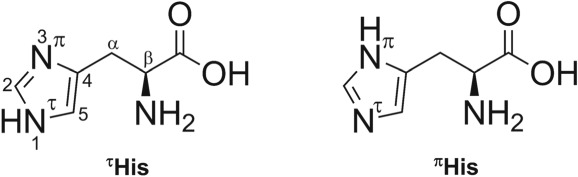
Molecular structures of two tautomeric forms of L‐histidine

Histidine (His; see Scheme 1) is an essential aromatic amino acid that is crucial for the biological functioning of proteins and enzymes. It is a precursor of the hormone histamine, an inflammatory agent in the immune response system, and a key residue in active sites of enzymes.^10^, ^11^ Due to the imidazole moiety, it can adopt two tautomeric conformations (Scheme 1) with H on either of the two N atoms referred to as the τ tautomer (^τ^His) and the π tautomer (^π^His), while it is suggested that the τ tautomer is preferred (80:20) in the gas phase at 298 K.^12^, ^13^

While the electron ionisation mass spectra of most of the amino acids are available,^14^ detailed knowledge on the ionisation energies (IE) of the molecules and the appearance energies (AE) of fragment ions formed upon ionisation are restricted to few amino acids investigated up to date. A literature survey reveals that the IEs and AEs were investigated experimentally for glycine, alanine,^15^ and valine.^15^, ^16^, ^17^ The vertical IE for His has been reported in the theoretical computational works of Huang et al^12^ and Close,^18^ who examined the vertical IEs of all common α‐amino acids. The formation of negative ions from amino acids upon resonant attachment of free electrons was previously studied as a function of the electron energy for glycine, proline, tryptophan, and methionine.^19^, ^20^, ^21^, ^22^, ^23^, ^24^, ^25^, ^26^, ^27^, ^28^ In contrast, for His, the electron energy dependence was neither studied nor discussed to the authors' knowledge. Voigt and Schmid studied negative ion mass spectroscopy of His in the gas phase,^29^ where they used a low‐temperature plasma source to generate low‐energy electrons with approximate kinetic energies between 2 and 4 eV. They reported the relative abundances of fragment anions like [His–COOH] ^−^ in the negative ion mass spectrum and obtained the dehydrogenated parent anion [His‐H]^−^ as the most abundant fragment anion.^29^ The electron energy dependence of negative ions formed via dissociative electron attachment (DEA) to His was only reported for the condensed phase by Abdoul‐Carime and Sanche.^30^ They measured the desorption of anions from thin films of His upon the impact of low‐energy electrons with kinetic energies between 5 and 35 eV.

In the present gas‐phase experiments, we investigate the formation of positive and negative ions of His in low‐energy electron collisions. The measurement of temperature dependent mass spectra at the electron energy of 70 eV shows an increase of mass peaks with temperature. Such behaviour was previously assigned to a fractional thermal decomposition of the His sample during the sublimation process. Therefore, we assign the ion yield of the parent cation and the dehydrogenated parent anion to form unambiguously from the nondecomposed sample. For other fragment anions and fragment cations, the possibility of formation from possible thermal decomposition products was considered. As will be shown below, the comparison of experimental data and the results of quantum chemical calculations allow a feasible assignment of most obtained ion yields to the intact neutral His precursor.

## 2 EXPERIMENTAL METHODS

### 2.1 Chemicals

**Figure 1:**
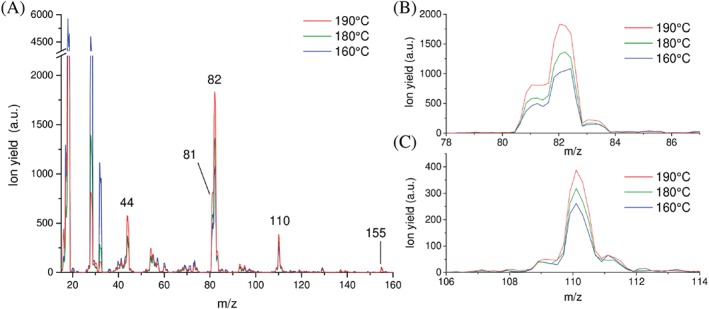
(A) Electron ionisation mass spectrum of the His sample heated to 160°C, 180°C, and 190°C, respectivly. The spectra at the two higher temperatures are normalised to the spectrum at 160°C in order to show same peak heights of the parent ion. Since the parent ion yield increases with temperature, constant background signals like N_2_^+^ and O_2_^+^ decrease in the spectra at elevated temperatures. (B) Detailed view of the spectrum shown in (A) in the mass region from *m*/*z* 78 to *m*/*z* 87. (C) Detailed view of the spectrum shown in (A) in the mass region from *m*/*z* 106 to *m*/*z* 114

His with stated purity of 99% was purchased from Sigma‐Aldrich and used as received. It appears as white powder under standard conditions. The vapour pressure of His required heating of the oven to about 160°C at around 10^−8^ to 10^−7^ mbar to allow the gas phase measurements with the utilised experimental set‐up. The temperature of the ion source was with 90°C always lower than the oven temperature, excluding subsequent effects at a later stage. Wilson et al^31^ performed VUV synchrotron ionisation studies of His and concluded that a fraction of His sample is already decomposed at the heating temperature of 100°C. Therefore, we also measured the temperature dependence of the electron ionisation mass spectrum at 70 eV in the course of the present experiments. The resulting mass spectra at three different temperatures of the sample are shown in Figure 1. The spectra are normalised to the ion yield of the parent ion at *m/z* 155. These data indicate that the most abundant ions above *m/z* 32 increase with oven temperature, as will be discussed in more details below.

### 2.2 Mass spectrometry experiments

The present experimental data were recorded with a crossed electron‐molecular beam set‐up combined with a quadrupole mass spectrometer. It was described in detail elsewhere.^32^ Briefly, electrons were emitted from a hairpin filament of the ion source and subsequently formed into an energetically and spatially focused beam by a hemispherical electron monochromator (HEM). A Faraday cup at the end of the HEM allows for checking stable electron beam conditions. The energy resolution for the present data was around 100 meV as determined from the full‐width‐half‐maximum (FWHM) of the well‐known SF_6_^−^/SF_6_ resonance.^33^ The molecular beam originates from an oven filled with His that was mounted perpendicularly to the HEM. It was heated at abovementioned conditions to evaporate the His. The gaseous compound was guided through a capillary with 1 mm inner diameter leaving it as effusive beam and crossing the electron beam in the interaction region at single‐collision conditions. Here, anions and cations were formed via dissociative electron attachment and electron ionisation, respectively. The ions were extracted by a weak electrostatic field into a quadrupole mass analyser. The mass selected ions were detected by a channel electron multiplier and processed by a discriminator and pulse counting unit.

For cations, ionisation and appearance energies were obtained by recording the ion efficiency curves of the mass‐selected ions. The electron energies were varied between 5 and 17 eV. The electron energy range was adapted for each cation to include the threshold and a region of about 3 eV below and above the threshold. The energy scale was calibrated by measuring the well‐known ionisation energy^34^ of Ne at 21.56 eV.

For negative ions, the anion efficiency curve of the mass‐selected products was obtained in the electron energy range of ~0 to 17 eV. The well‐known resonance position at 0 eV of SF_6_^−^/SF_6_ 33 was deployed for calibration of the electron energy scale.

## 3 COMPUTATIONAL METHODS AND DATA ANALYSIS

### 3.1 Determinations of IEs/AEs

The behaviour of ionisation cross sections in the threshold region was first described by Wigner and later extended by Wannier. Wigner theoretically developed a simple power law, where the shape of the cross‐section close to threshold depends on the number of outgoing electrons.^35^ In case of single ionisation by impact of an electron, he predicted a linear behaviour of the cross section. Wannier extended the theory for electron ionisation processes leading to a three charged particles final state (two electrons and one ion),^36^ where such developed model describes the cross‐section behavior close to the threshold. Including a Heaviside function θ*,* the cross‐section *σ* can be expressed as follows:

(1) $$f\left(E\right)=b+c\;{\left(E-\mathrm{AE}\right)}^{n}\theta \left(E-\mathrm{AE}\right),$$

where *b* is a linear background, *c* is a scaling parameter, *E* is the electron energy, *AE* is the appearance energy, and *n* is the exponent. Wannier calculated *n* only for hydrogen^36^ and must otherwise thus be determined experimentally.

The experimental set‐up entails a finite energy resolution in form of a Gaussian distribution

(2) $$g\left(E\right)=\frac{1}{\sqrt{2\pi \rho }}\exp\left(-\frac{E^{2}}{2\rho^{2}}\right)$$

with the standard deviation *ρ* representing the resolution. The cross‐section function is convoluted with the Gaussian profile giving the prediction for an experimentally measured cross‐section curve with resolution *ρ*

(3) $$\begin{array}{l} \sigma \left(E\right)=\left(f*g\right)\left(E\right)=\int_{-\infty }^{\infty }d\mathrm{xf}\left(x\right)g\left(E-x\right)=\int_{-\infty }^{\infty }dx\left[b+c{\left[E-\mathrm{AE}\right]}^{n}\theta \left[E-\mathrm{AE}\right]\right]\frac{1}{\sqrt{2\pi \rho }}\\ \exp\left(-\frac{{\left(E-x\right)}^{2}}{2\rho^{2}}\right)=b+\frac{c}{\sqrt{2\pi }}\;\rho^{n}\Gamma \left(n+1\right)\;\exp\left(-\frac{1}{4\rho^{2}}{\left(\mathrm{AE}-E\right)}^{2}\right)\mathrm{Dn}+1\left(\frac{1}{\rho }\left(\mathrm{AE}-E\right)\right), \end{array}$$

where *Γ* is the gamma function and *D*_n+1_ is a parabolic cylinder function.

This function for the cross‐section allows fitting the experimental data. As a result, the parameters of such function, especially the *AE*, can be extracted. For this purpose, a software tool was developed based on a previous version^37^ written in Python,^38^ using the SciPy,^39^ NumPy,^40^ and Matplotlib^41^ libraries. The cross‐section function is fitted by means of the Chi‐Square (*χ* ^2^) method and minimising the squared sum of the difference of data and fit for each point. The data points are weighted according to their respective uncertainties. The required input parameters are the fitting range around the estimated *AE* and the resolution *ρ*. The number of iterations depends on the number of data points of the according measurement and is higher for larger data sets. With each iteration of the fitting procedure, the fitting range is narrowed and centred around the determined *AE* value, until either the set number of iterations is reached or alternatively the change of the fitting range falls below a beforehand specified value. It has proven beneficial for the procedure to initially set a higher resolution than experimentally achieved and subsequently reduce it to its actual value throughout the iterations. Besides the *AE* value, the output parameters include the linear background *b*, the scaling factor *c*, and the exponent *n*. Errors resulting from the fit can be extracted from the covariance matrix. A further uncertainty arises from the setting of the fitting range. We conducted test series and found that the determined onset value remains stable within maximal deviations of 50 meV for fitting ranges ≥3 eV, which was then chosen as standard input value. Thus, the statistical uncertainty on an AE value consists of the uncertainty arising from the fit and the uncertainty caused by the choice of the fitting range. For the analysis, the square of their quadratic addition is stated.

Since the energy scale was calibrated with this analysis tool, the uncertainty on the AE of the neon ion yield transfers into a systematic uncertainty of all analysed cations and amounts to 10 meV. Since the value is equal for all cations, it will not be stated during the results part for reasons of clarity. It is important to note that the energy resolution of the HEM and the error on the *AE* value are two different parameters.

For channels suggesting two or more appearance energies, the number of thresholds is a further input parameter for the programme. The first onset is fitted as described above, and subsequently, the fit is subtracted from the data and the same method is applied on the next threshold. The defined fitting ranges may each only include one threshold.

### 3.2 Determination of peak onsets in anion efficiency curves

For data analysis of the DEA processes, (multiple) Gaussian fits were applied to the experimental data curves. The peak maxima *x*_*max*,*i*_ of each *i*‐th peak was taken from the fit together with the error. To obtain the onsets of the reaction, it has to be taken into account that the resolution of the set‐up broadens the peak by introducing a natural tail. Earlier methods^42^, ^43^ applied linear fits to the steepest part of the Gaussian profile which resulted in large uncertainties in the onset. Here, a new method is introduced. The onset is defined as follows:

(4) $$x_{\text{onset}}=x_{\max,1}-2\sigma ,$$

with *x*_*max*,1_ the peak maximum and *σ* the standard deviation of the first peak. This cuts the outbounding tail at a defined position, making the method both robust concerning uncertainties and reproducible. Two *σ* were chosen as the tail is cut at a comparable level to earlier methods.

### 3.3 Quantum chemical calculations

The lowest energy structures of His tautomers, τ (^τ^His) and π (^π^His), were taken from the study of Stover et al.^13^ Quantum chemical calculations employing M062x/aug‐cc‐pVTZ level of theory^44^, ^45^ and basis set^46^, ^47^ were carried out to calculate ionisation energies, adiabatic electron affinities, and the free energy of reactions, Δ*G*(298K), which is calculated for each fragmentation pathway as Δ*G* = Σ*G* (products) − Σ*G* (reactants).^48^ Calculated frequencies confirmed that the structures are local minima on the potential energy surface and not transition states. We estimate an error of less than 0.09 eV for the reaction energies and 0.11 eV for ionisation potentials from the reported mean unsigned error for M062x thermochemistry and ionisation potentials, respectively.^44^ We have carried out some computations with the DSD‐PBEP86 double‐hybrid DFT method,^49^ which performs well for a wide range of chemical properties.^50^ The results show that M062x compares favourably to this higher level method, which lends support to the use of M062x more generally for the present set of systems.

The thermodynamic threshold for the DEA reactions discussed in the next section can also be expressed by Δ*G*([M‐X]^−^) = DE(M‐X) – AEA(M‐X), where M denotes the molecule and X the released neutral, DE(M‐X) is the bond dissociation energy and AEA(M‐X) is the adiabatic electron affinity of the corresponding product. The threshold energy for the experimental observation of [M‐X]^−^ in electron attachment experiments coincides with Δ*G*([M‐X]^−^), if the products are formed with no excess energy. Otherwise, fragmentation reactions occur at electron energies above the thermodynamic threshold Δ*G*([M‐X]^−^) carrying away the excess energy as kinetic energy or internal energy, which can induce further fragmentation.

All calculations were performed with the Gaussian 09 programme,^51^ and the structures and dissociation reactions considered in this study are summarised in the Supporting Information.

## 4 RESULTS AND DISCUSSION

### 4.1 The mass spectrum at ~70 eV and electron ionisation close to threshold

The mass spectrum shown in Figure 1A reveals that the parent ion His˙^+^ at *m/z* 155 is a minor species among the other ions observed. Therefore, one may conclude that strong fragmentation may occur upon ionisation of His by electron interactions. The most abundant peaks above *m/z* 32 can be found in the present spectrum at *m/z* 110 ([His–COOH]^+^), *m/z* 82 ([C_4_N_2_H_6_]˙^+^), *m/z* 81 ([C_4_N_2_H_5_]^+^), and *m/z* 44 (CO_2_^+^/[NH_3_CHCH_2_]˙^+^). Major peaks at low masses, *m/z* 18, 28, and 32, can be assigned to ionisation of residual water and air. The electron ionisation mass spectrum (at 75 eV) reported in the Spectral Database for Organic Compounds^52^ is in qualitative agreement with the present spectrum, since the same abundant ions—with *m/z* 82 and *m/z* 81 as the most abundant species—are observed. However, the relative abundance of the parent ion in Matsuyama and Wasada^52^ is lower compared with the present spectrum and a peak at *m/z* 28 (with ~20% abundance compared with the major ion yield found at *m/z* 82) is also visible in the spectrum of Matsuyama and Wasada^52^ which—in the absence of the O_2_^+^ peak at *m/z* 32—can be likely ascribed to CO^+^. The current spectrum does not allow a conclusion on the ion yield at *m/z* 28, since it is contaminated by abundant N_2_^+^ background signal. Another disagreement can be found for the peak at *m*/*z* 44. This peak shows a minor yield in Matsuyama and Wasada^52^ but an abundant peak at *m/z* 44 is found in the present spectrum. The deviations may be ascribed to the different temperatures used, which will influence the yields of the various ionic products. The spectrum shown in Matsuyama and Wasada^52^ was recorded at the sample temperature of 190°C (a spectrum at the same sample temperature is included in Figure 1) while the ion source temperature of 230°C was considerably higher than the presently used 90°C.

We note that electron^53^ and photon^54^ ionisation studies with His were also carried by means of a more gentle sublimation method, respectively. Both studies employed laser‐induced acoustic desorption (LIAD) of the His sample, where thermal decomposition should not play a considerable role. Interestingly, the relative ion yield of the His cation in the mass spectrum was higher in case of ionisation by intense femtosecond laser pulses then in the LIAD/electron ionisation experiment. Overall, above *m/z* 44 good agreement is obtained for the present spectrum and the spectra reported in Jarrell et al^53^ and Duffy et al,^54^ ie, by this comparison, it may be concluded that the major fragment ion peaks at *m/z* 54, 81, 82, and 110 arise from ionisation of intact His.

Yet in contrast, Wilson et al assigned the ion yield at *m/z* 82 to result from ionisation of thermal decomposition products,^31^ where thermal decomposition was already operational at the His sample temperature of 100°C; however, we note that they produced gas‐phase His by particle evaporation. Interestingly, they also observed a peak at *m/z* 111, which was about 1.5 times more abundant than *m/z* 110. These authors proposed that His loses CO_2_ by thermal decomposition and the ion yield of [His‐CO_2_]^+^ at *m/z* 111 is formed by nondissociative photon ionisation of neutral histamine ([His‐CO_2_]). The release of neutral CO_2_ as one of the gaseous products (besides H_2_O and NH_3_) upon thermal decomposition of His was observed by Weiss et al,^55^ while Smith et al^56^ suggested the abundant formation of imidazole (mass 68 u) upon pyrolysis of His. In the current mass spectra, we obtain only a very weak signature of the imidazole ion compared with CO_2_. The present mass spectrum also does not provide evidence of abundant formation of ([His‐CO_2_]^+^ like in Wilson et al,^31^ where the latter fragment ion was more abundant than [His–COOH]^+^. The present ratio of [His‐CO_2_]^+^ and [His–COOH]^+^ is only ~20% at the sample temperature of 160°C and thus just a factor of ~3 above the isotope ratio of 6.6% due to C‐13, N‐15, or H‐2 in [His–COOH]^+^. The absence may be ascribed to the different nature of the ionising particle—electrons in the present work against photons in Wilson et al^31^—or the subsequent degradation of [His‐CO_2_] due to the elevated temperature in the present experiment. We favour the latter explanation since the electron ionisation mass spectrum of histamine available at the NIST database^14^ shows an abundant peak at *m/z* 30 ([NH_2_CH_2_]^+^) which is by a factor of ~10 stronger than the parent ion. Based on this ratio, the contribution of the histamine parent ion to the signal *m/z* 111 would be only ~2% due to the low abundance *m/z* 30 in the present mass spectrum at 160°C. Therefore, we rule out that the neutral gaseous beam contains considerable amounts of histamine and assign the ion signal at *m/z* 111 to be mainly formed from the isotope contribution of [His–COOH]^+^ and background signal. This assignment is further supported by the constancy of the ion signal at *m/z* 111 when the sample temperature is increased (see Figure 1C). This means that the increase of the [His–COOH]^+^ isotope and the decrease of the background signal, both relative to the signal of the parent ion, keep their balance with increasing temperature.

**Figure 2:**
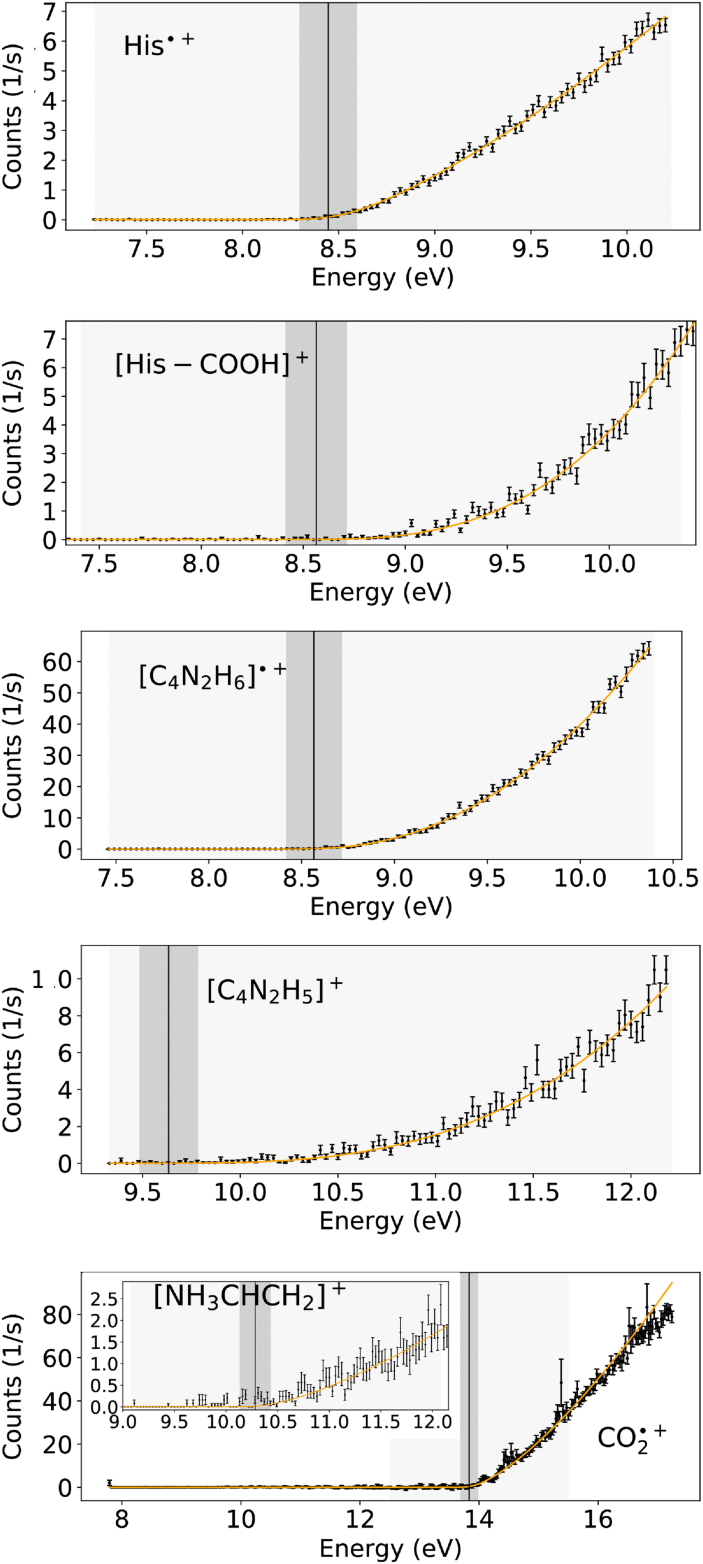
Ion yield curves close to threshold of the experimentally observed cations. The black dots and error bars represent the data and the orange line is the fitted Wannier function convoluted with a Gaussian profile. The vertical black lines show the appearance energy with the energy resolution of the HEM marked as dark grey region. Fitting regions are represented by the light grey areas. The lowest panel associated with *m*/*z* 44 shows a magnification of the first threshold region. The inset displays the whole measurement range including the second threshold at higher energies [Colour figure can be viewed at http://wileyonlinelibrary.com]

**Figure 3:**
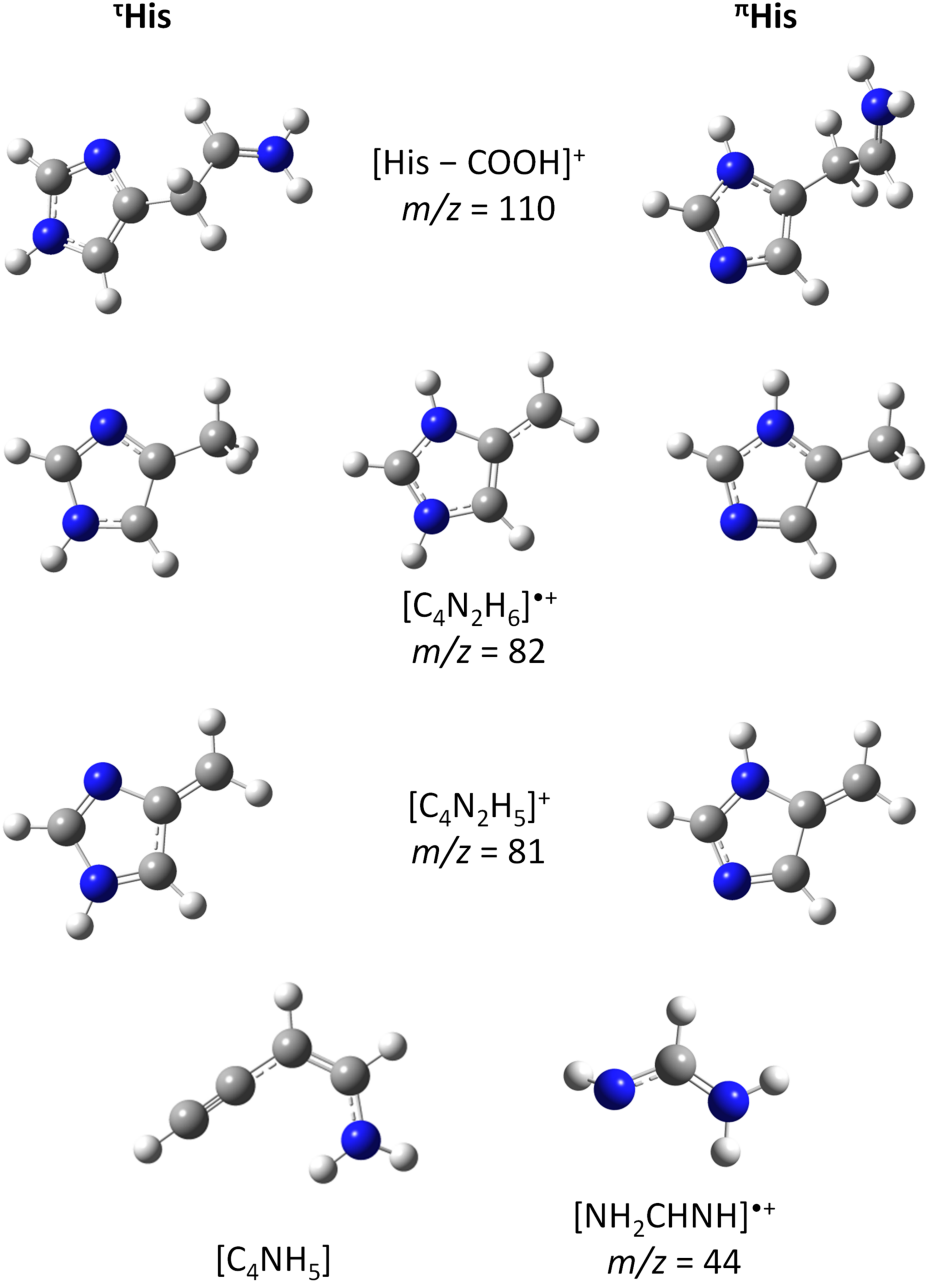
M062x/aug‐cc‐pVTZ calculated minimum energy structures of cations and neutrals formed upon electron ionisation of the tautomer ^τ^His (left) and ^π^His (right), respectively [Colour figure can be viewed at http://wileyonlinelibrary.com]

Figure 2 shows the ion yield curve close to the threshold for all investigated cations as a function of the electron energy. The experimentally determined IE of His and the derived AEs of ions found at *m/z* 110, 82, 81, and 44 are summarised in Table 1. The corresponding calculated structures of the cations are shown in Figure 3 for both tautomers. Table 2 summarises for all observed cations the exponent of the fitted Wannier function.

**Table 1:** Summary of the observed cations including their mass, the composition of the products, and the according neutrals and ionisation/appearance energy

Mass (*m/z*)	Cation	Neutral(s) in Calc.	Ionisation/Appearance Energy, eV			Lit
			Exp.	Calc.^a^		
				^τ^His	^π^His	
155	His˙^+^	**‐**	8.40 ± 0.04	8.86/8.30 (VIE/AIE) 8.88/8.33^a^	8.45/8.13 (VIE/AIE) 8.44/8.13^a^	8.2‐8.72^d^
110	[His–COOH]^+^	CO_2_ + H˙	8.52 ± 0.08	8.53	8.98	8.5^f^
82	[C_4_N_2_H_6_]˙^+^	CO_2_ + H_2_ + HCN	8.54 ± 0.04	8.62^b^	8.51^b^	‐
				7.97^c^	7.90^c^	
81	[C_4_N_2_H_5_]^+^	CO_2_ + H_2_ + H_2_CN˙	9.56 ± 0.11	9.73	9.67	‐
44	[NH_3_CHCH_2_]^+^	[C_3_N_2_H_3_]˙ + CO_2_	10.23 ± 0.09	10.41	10.34	‐
44	CO_2_˙^+^		13.8 ± 0.5	13.94		13.78^e^

*Note*. Experimental and calculated values are stated along with data available from literature. Uncertainties of the experimental values refer to the statistical error. The systematic uncertainty is equal for all and amounts to 10 meV.

M062x/aug‐cc‐pVTZ calculated ionisation potentials and free energies of reactions Δ*G*(298K).

a Vertical and adiabatic ionisation energies obtained by DSD‐PBEP86 double‐hybrid DFT method.

b Values are associated with reaction (6a) and 4‐methylimidazole ion.

c Values are associated with reaction (6a) and 4‐methylene‐imidazole ion.

d Refer to previous works.^12^, ^18^, ^31^

Refer to Wilson et al.^31^

e Refer to Linstrom and Mallard.^14^

**Table 2:** Summary of the observed cations including their mass and the exponent of the Wannier function as fitted with the analysis tool

Mass, *m*/*z*	Cation	Exponent *n*
155	His˙^+^	1.33 ± 0.04
110	[His–COOH]^+^	2.8 ± 0.2
82	[C_4_N_2_H_6_]˙^+^	2.07 ± 0.06
81	[C_4_N_2_H_5_]^+^	2.9 ± 0.3
44	[NH_3_CHCH_2_]^+^	1.5 ± 0.2
44	CO_2_˙^+^	1.4 ± 0.1

*Note*. Uncertainties refer to the error resulting from the fit.

In the present experiments, the experimentally measured IE of His is (8.40 ± 0.04) eV, which is in good agreement with the calculated vertical ionisation energy (VIE) of 8.45 eV of ^π^His. For the ^τ^His tautomer, we obtain a calculated VIE of 8.86 eV. The ^π^His is 0.08 eV above the ground state of ^τ^His, which is the most stable tautomer. The population ratio at the approximate evaporation temperature of 440 K is 1:0.12. However, we do not observe a clearly discernible threshold close to 8.86 eV for the ^τ^His fraction in the neutral molecular beam. The calculated adiabatic ionisation energies (AIEs) for His tautomers are 8.13 eV for ^π^His and 8.30 eV for ^τ^His. Thus, our experimental value is also close to the AIE of ^τ^His tautomer. We have performed computations with the DSD‐PBEP86 double‐hybrid DFT method that confirmed the values of VIE and AIE obtained by M062x (see Table 1). We have to note that the VIEs and AIEs of both tautomers are very sensitive to the conformations as it was observed by Huang et al who performed full conformational search and suggested that the equilibrium population should lead to the observable ionisation energy of 8.5 eV.^12^ The reported VIEs for the most stable conformers (within 0.1 eV) for ^π^His range between 8.14 and 8.52 eV, while for ^τ^His, it ranges between 8.43 and 8.60 eV.^12^ For the most stable conformers, the VIEs are 8.43 and 8.52 eV for ^τ^His and ^π^His, respectively. Wilson et al^31^ reported an ionisation energy of (8.2 ± 0.1) eV, which was obtained for VUV photon ionisation experiments. They also calculated ab‐initio structures of seven low‐lying conformers of ^π^His and their cations and derived VIEs and AIEs. They suggested that the presence of up to five conformers led to smearing out the photoionisation onset. Using a heater temperature of 100°C, these authors ascribed the experimentally derived IE to the calculated adiabatic values of the second and third most energetically favourable conformer. The adiabatic values of 8.16 eV were in good agreement with their experimental result and the presently calculated AIE of ^π^His. However, they have only considered ^π^His and only several conformers. Huang et al^12^ reported for the most stable conformers the AIEs of 7.92 eV for ^τ^His and 8.17 eV for ^π^His, where the AIE of ^π^His is in better agreement with the experiment of Wilson et al.^31^ As already mentioned, Wilson et al produced gas‐phase His by particle evaporation, where particles were produced by atomising solutions of His in water that could change the tautomeric population. As far as other theoretical calculations in the literature are concerned, Gil et al^57^ calculated the AIEs of different amino acids and obtained a value of 7.88 eV for His, while in the work of Close,^18^ the VIEs were reported for different conformers and different levels of theories ranging between 8.49 and 8.72 eV. Noteworthy, the presently measured ionisation energy of His (8.40 ± 0.04) eV is close to the reported experimental VIE of imidazole and 1‐methyl‐1*H*‐imidazole^58^, ^59^ of 8.66 eV, which suggests that the electron is removed from imidazole moiety.

Even though our experimental ionisation energy of His is in great agreement with the calculated VIE of 8.43 eV reported by Huang et al for ^τ^His tautomer,^12^ due to the likely presence of variety of conformations and the close value of the VIE for the ^π^His (8.52 eV), it is difficult to make a conclusive assignment on whether the experimental threshold corresponds to ionisation of ^τ^His or ^π^His.

The presently observed ion yields of fragments may result through the following reactions:

(5) $$m/z=110:e^{-}+\mathrm{His}\to {\left[\mathrm{His}-\text{COOH}\right]}^{+}+{\mathrm{CO}}_{2}+H^{\cdot }+2e^{-}$$

(6a) $$m/z=82:e^{-}+\mathrm{His}\to {\left[C_{4}N_{2}H_{6}\right]}^{\cdot +}+{\mathrm{CO}}_{2}+H_{2}+\mathrm{HCN}+2e^{-}$$

(6b) $$m/z=82:e^{-}+\left[C_{4}N_{2}H_{6}\right]\to {\left[C_{4}N_{2}H_{6}\right]}^{\cdot +}+2e^{-}$$

(7a) $$m/z=81:e^{-}+\mathrm{His}\to {\left[C_{4}N_{2}H_{5}\right]}^{+}+{\mathrm{CO}}_{2}+H_{2}+H_{2}{\mathrm{CH}}^{\cdot }+2e^{-}$$

(7b) $$m/z=81:e^{-}+\left[C_{4}N_{2}H_{6}\right]\to {\left[C_{4}N_{2}H_{5}\right]}^{+}+H^{\cdot }+2e^{-}$$

(8a) $$m/z=44:e^{-}+\mathrm{His}\to {\left[{\mathrm{NH}}_{2}\text{CHNH}\right]}^{\cdot +}+\left[C_{4}{\mathrm{NH}}_{5}\right]+{\mathrm{CO}}_{2}+2e^{-}$$

(8b) $$m/z=44:e^{-}+{\mathrm{CO}}_{2}\to {\left[{\mathrm{CO}}_{2}\right]}^{\cdot +}+2e^{-}$$

The experimental threshold for the product ion [His–COOH]^+^ at *m/z* 110 is (8.52 ± 0.08) eV (Table 1). This fragment ion may be formed from His through a simple bond cleavage releasing CO_2_ + H˙, see reaction (5) and Figure 3. The calculated bond dissociation energy is similar for the ^τ^His and the ^π^His tautomers (3.27 and 3.19 eV, respectively). The calculated free energy of reaction (5) is 8.53 eV for ^τ^His and 8.98 eV for ^π^His, which is in excellent agreement with the experiment suggesting the ^τ^His tautomer present in the beam. We have noted that after the His–COOH bond dissociation, the strong interaction between the H of the amino group with N of the imidazole ring can lead to the H transfer towards imidazole resulting in structures that are about 0.5 eV lower in energy (see Supporting Information). Wilson et al concluded in their photon ionisation studies of thermally sublimated His at 363 K that [His–COOH]^+^ is indeed a fragment formed by dissociative ionisation of His and excluded thermal decomposition products as neutral precursors of this ion. The present mass spectra at different oven temperatures show an increase of the [His–COOH]^+^ signal by a factor of about 1.5 %, when the temperature is increased from 160°C to 190°C. We ascribe this effect rather to the increase of internal energy in the His precursor instead of the increase in thermal decomposition. The alteration of mass spectra due to the increase of molecular temperature was previously reported for various molecules.^60^ We further note that neutral [His–COOH]˙ is a radical, and one may expect that even electron species are formed by thermal decomposition.

The fragment ion [C_4_N_2_H_6_]˙^+^ at *m/z* 82 has a measured appearance energy of (8.54 ± 0.04) eV, which is in fair agreement with the calculated AE of 8.62 eV for ^τ^His 8.51 eV for ^π^His, where the 4‐methylimidazole ion is formed with concomitant breakage of the whole chain into simple molecules CO_2_ + H_2_ + HCN, see reaction (6a). Simple C_α_–C_β_ bond cleavage with transfer of H from C_β_ or from the amino group to C_α_ during dissociation would require energies far above the experimentally obtained AEs (see Supporting Information). If one follows the assignment by Wilson et al,^31^ (signal at *m/z* 82 would be from a thermal decomposition product identified as 4‐methylimidazole), reaction (6b) may occur and the AE should be compared with the IE of neutral [C_4_N_2_H_6_]. The calculated VIEs are 8.64 eV (imidazole ring from ^τ^His) and 8.61 eV (from ^π^His) and thus above the experimental value. The reaction (6b) related to the thermal decomposition can be ruled out following the argumentation above and below on the discussion of the formation of ion at *m/z* 81.

From the theoretical point of view, formation of the fragment ion [C_4_N_2_H_6_]˙^+^ received particular attention in literature,^61^, ^62^, ^63^ since it is the most abundant ion in the mass spectrum of His and corresponds to the charged organic substituent [RH]˙^+^ in general consideration of the amino acid structures [R−CH (NH_2_)COOH]. In previous computational studies, it was suggested that a proton transfer from the amino group to basic nitrogen of the imidazole moiety occurs after ionisation of His and in the following step, the C_α_−RH^+^ bond is broken. Gil et al^61^ calculated the electronic energy profile for such fragmentation process for ^τ^His and obtained a height barrier of 0.80 eV relative to the ionised ^τ^His. In this case, the formed ion is the 4‐methylene‐imidazole ion (see Figure 3) that is 0.68/0.65 eV more stable over the 4‐methylimidazole ion (related to ^τ^His and ^π^His, respectively). However, the calculated free reaction energy for this process is 8.21 eV for ^τ^His and 8.14 eV for ^π^His and accounting for the barrier by Gil et al of 0.73 eV (the difference between the formed products and the barrier height) would result in AEs that are above the measured AE (see Supporting Information). If the chain would decompose as in (6a), the calculated free reaction energy is 7.97 eV for ^τ^His and 7.90 eV for ^π^His, where accounting for the barrier (0.73 eV) by Gil et al would give 8.7 and 8.63 eV, respectively, which is also above the measured AE value. Thus, we can conclude that the formed ion at *m/z* 82 is related to 4‐methylimidazole, and our data suggest that [C_4_N_2_H_6_]˙^+^ is resulting from ionisation of the intact His molecule.

Another abundant fragment ion is [C_4_N_2_H_5_]^+^ at *m/z* 81, where the backbone of the amino acid is broken into several simple molecules (reaction (7a)) with free reaction energies of 9.73 eV for ^τ^His and 9.67 eV for ^π^His, which would be in good agreement with the experimental threshold of (9.56 ± 0.11) eV (see Table 1). Simple C_α_−C_β_ bond cleavage showed to have free reaction energies which are lower than the experimental values, namely, 8.99 eV for ^τ^His and 8.93 eV for ^π^His and can be excluded since no threshold at these energies is discernible in the present experimental data. Since the formation of 4‐methylimidazole by thermal decomposition was suggested in Wilson et al,^31^ we also investigated if the ion yield at *m/z* 81 was formed by hydrogen loss from 4‐methylimidazole, reaction (7b). The mass spectrum of 4‐methylimidazole in the NIST database suggests this process, since the ratio of *m/z* 81 to *m/z* 82 is 0.75. We calculated the corresponding AEs, obtained a value at 10.5 eV considering that the ion yield at *m/z* 81 is formed by the loss of an H atom from the CH_3_ group of 4‐methylimidazole and the AE is 12.7 eV if the H atom is lost from the N position. The disagreement to the experimental values supports the conclusion from the mass spectra discussed above (*m/z* 82 is a fragment ion from ionised His), and therefore, we assign the experimentally found threshold of (9.56 ± 0.11) eV to dissociative ionisation of His.

The fragment ion at *m/z* 44 exhibits two clearly visible thresholds in the ion efficiency curve (see Figure 2). We found the first threshold at (10.23 ± 0.09) eV and the second at (13.8 ± 0.5) eV. It should be noted that the ion yield associated to the first threshold is much less abundant than the ion yield rising above the second threshold. The uncertainty of the second threshold is larger than all the other reported uncertainties since the ion yield assigned to the first threshold causes an additional background for the analysis. The present quantum chemical calculations show that the first threshold may be assigned to formation of [NH_2_CHNH]˙^+^ (see reaction 8a and Figure 2) formed upon electron ionisation of His. Assuming the neutrals [C_4_NH_5_] + CO_2_ formed in the fragmentation process, the corresponding free reaction energies of 10.41 eV for ^τ^His and 10.34 eV for ^π^His are in fair agreement with the first experimental threshold. The second threshold at (13.8 ± 0.5) eV is likely due to the ionisation of neutral CO_2_ formed by thermal decomposition of His, reaction 8b. The calculated IE value (13.94 eV) is only slightly higher than the experimental IE of 13.78 eV reported on the NIST homepage.^14^ We also investigated computationally if CO_2_˙^+^ may be formed upon dissociative ionisation of His. Since the free reaction energies for CO_2_˙^+^ formation from His is 13.45 eV for ^τ^His and 13.33 eV for ^π^His, we can exclude this process. Further breaking of the neutral fragment would require more than 14.10 eV.

As noted above, from the experimental ionisation energy of His it is not obvious, whether ^τ^His or ^π^His is present in the beam. When comparing the calculated thresholds for the two tautomers (see Table 1) leading to the formation of the fragments of *m/z* 44, 81, and 82, within the error of the calculation (0.09 eV) the ^τ^His and ^π^His are indistinguishable. The only channel for which ∆*G* differs for the two tautomers by ~0.5 eV is the formation of the *m/z* 110, which suggests the presence of the ^τ^His in our experiment.

### 4.2 Dissociative electron attachment

**Figure 4:**
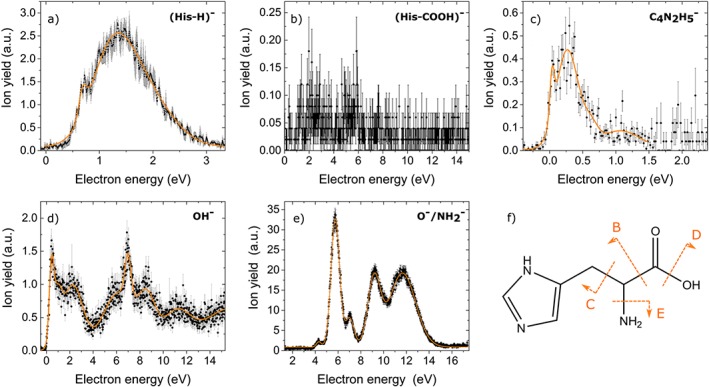
Ion yields (black dots and error bars) and cumulative multiple asymmetric Gaussian fit (orange line) of the experimentally observed dissociation channels. The ion yields are in arbitrary units but the relative height is comparable among all anions. F: Molecular structure of L‐histidine with marked suggestions of strand breaks for the dissociation channels B‐E [Colour figure can be viewed at http://wileyonlinelibrary.com]

**Figure 5:**
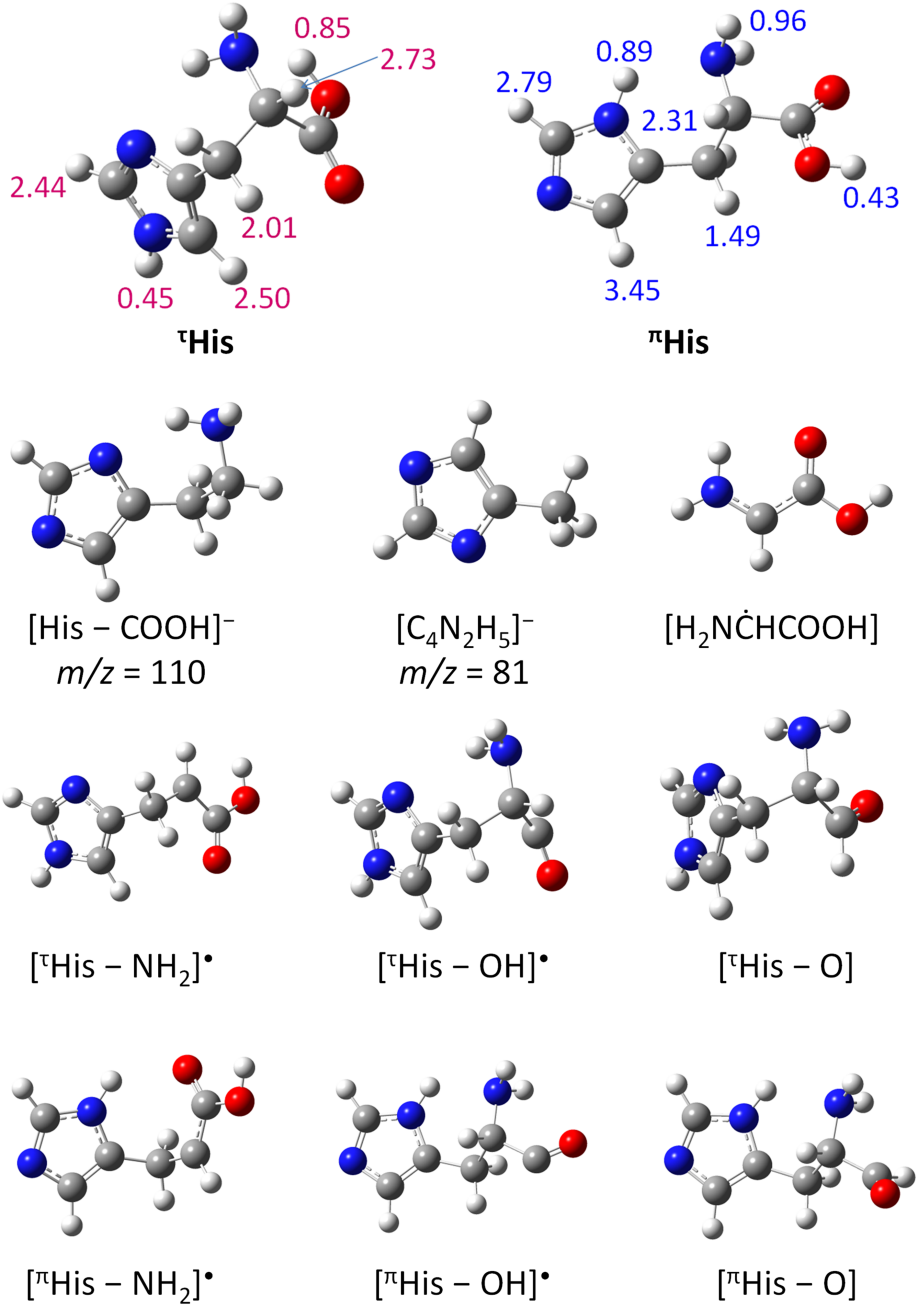
M062x/aug‐cc‐pVTZ calculated minimum energy structures of anions and neutrals formed upon DEA to neutral His. The top of the figure shows values of free energy of reaction (9) related to the release of H^•^ radical from different sites of His. The value of 2.73 eV refers to the removal of H˙ radical from C_β_ position [Colour figure can be viewed at http://wileyonlinelibrary.com]

In the course of the present experiments on negative ion formation, anion yields were obtained at *m/z* 154 ([His–H]^−^), at *m/z* 110 ([His–COOH]^−^) at *m/z* 81 ([C_4_N_2_H_5_]^−^), at *m/z* 17 (OH^−^), and at *m/z* 16 (O˙^−^/NH_2_^−^). Figure 4 shows the ion yields as a function of the incident electron energy together with cumulative multiple fits of the experimentally observed peak structures. The anion assignment and the composition of the corresponding neutral products for each reaction channel are summarised in Table 3 together with the measured peak positions. The respective structures of anions and neutral products are shown in Figure 5. The dehydrogenated parent anion is formed in the following DEA reaction upon electron attachment to His:

(9) $$e^{-}+\mathrm{His}↔{\left({\mathrm{His}}^{\cdot -}\right)}^{\#}\to {\left[\mathrm{His}‐H\right]}^{-}+H^{\cdot },$$

**Table 3:** Summary of all observed anions including their mass, the composition of the charged and neutral products, peak positions, and thresholds for the reaction determined experimentally, by calculations and compared with literature values

Mass (*m/z*)	Anion	Position of Resonances, eV							Threshold, eV			Neutral(s) in Calc.
									Exp.	Calc.^a^		
										^τ^His	^π^His	
154	[His–H]^−^	0.73	1.33						0.4 ± 0.1	0.45	0.43	H˙
110	[His–COOH]^−^	**‐**							**‐**	0.61	0.55	CO_2_ + H˙
81	[C_4_N_2_H_5_]^−^	0.05	0.27	1.07					0.00 ± 0.01	−0.31	−0.37	H_2_NĊHCOOH
17	OH^−^	0.47	1.00	2.29	6.25	7.00	8.50	11.31	0.04 ± 0.03	2.75	2.63	[His–OH]˙
										2.44	2.37	[His–COOH]˙ + CO
16	O˙^−^	4.30	7.00	9.20	11.68				3.9 ± 0.1	2.61	2.31	[His–O]
	NH_2_^−^	5.74								5.56	5.72	[His – NH_2_]˙
										3.65^b^	3.65^b^	

*Note*. The uncertainty of the peak positions amount to ≤0.05 eV resulting from the Gaussian fit and stepwidth set. The uncertainty stated for the experimentally derived threshold relates to the fit.

a M062x/aug‐cc‐pVTZ calculated free energies of reactions Δ*G*(298K).

b Values require not just a simple bond cleavage but also rearrangement to form the most stable neutral shown in Figure 5.

where (His˙^−^)^#^ denotes the temporary negative ion (TNI) formed by initial resonant capture of a free electron. In general, the TNI may undergo dissociation into various stable anions, where the excess charge is retained, and including at least one neutral (fragment) formed. However, auto‐detachment is also in competition with DEA, where the excess charge is spontaneously emitted from the TNI. The ion yield of [His–H]^−^ is shown in Figure 4A) and exhibits two peaks at 0.73 eV (with the experimental threshold of (0.4 ± 0.1) eV) and 1.33 eV. Dehydrogenation, defined here as the loss of a single hydrogen, can arise through the emission of H˙ from any of the C, N, or O sites. Quantum chemical calculations show that the free energy of reaction (9) with the loss of H˙ from the −COOH group is 0.85 eV for ^τ^His and 0.43 eV for ^π^His (see Figure 5, where the free energies for hydrogen loss from all possible sites are shown). However, the removal of H˙ from the N positions of imidazole moiety or −NH_2_ group are also low in energy in the range of 0.43 to 0.96 eV. Removal of H˙ from the C positions is least preferred requiring at least 1.49 eV in the case of C_α_, in other instances ranging from 2.01 to 3.45 eV. The adiabatic electron affinity (AEA) of [His–H]˙ is in some cases relatively high, eg, for the fragment, which lost the H˙ from the −COOH group, the AEA amounts to 3.74 eV, or for the loss of H˙ from the N position of the imidazole ring and −NH_2_ group it is ~3 eV for both tautomers.

The dehydrogenation reaction upon DEA to amino acids is often an abundant channel and was therefore observed in linear amino acids like for example glycine,^19^ alanine,^64^ methionine,^23^ and aromatic amino acids like proline.^65^, ^66^ Early DEA studies with formic acid^67^ as a model molecule for amino acids suggested that hydrogen loss from the carboxyl group proceeds through electron attachment into the π^*^ orbital of the −COOH group. As the position of the observed resonances and the shape of the ion yields from the amino acid is identical to the one observed for formic acid HCOOH, this suggestion was tentatively used also for amino acids.^19^ However, more recent experimental^68^ and theoretical studies^69^ favoured a direct mechanism with attachment of the excess electron into the σ^*^ (OH) orbital. In all previous cases where the anion yield was measured as a function of the electron energy, the ion yield had a characteristic shape with an abrupt steep onset at ~1 eV. In contrast, in the present case of His, the ion yield has a notably very different shape and the first peak observed is at lower energy, ie, 0.73 eV. Moreover, it is also not the most abundant anion among the observed fragment anions in contrast to other amino acids. Interestingly, in DEA to His the shape of the ion yield of [His–H]^−^ shown in Figure 4A rather resembles the shape of the dehydrogenated anion formed in electron attachment to gas‐phase imidazole,^70^ but it is shifted here by about ~1 eV towards lower energies. In the case of imidazole, it was suggested that the electron attachment takes place through the π^*^ resonance that couples to a dissociative σ^*^ state localised at the N−H bond.^70^ Such is only possible if the nuclear wave packet survives long enough to allow the system adiabatic crossing between states. According to the quantum chemical calculations, the removal of H^•^ from the N position of the imidazole moiety of His requires at least 0.45 eV. It is therefore indeed energetically possible that the observed peak structure results from H‐loss from the imidazole. However, also H‐loss from the COOH group may occur as well.

As mentioned in the Introduction section, Voigt and Schmidt studied negative ion mass spectroscopy of His in the gas phase.^29^ They obtained [His–H]^−^ as the most abundant anion for low‐energy electrons with approximate kinetic energies between 2 and 4 eV. In contrast to the present single collision conditions, they used higher pressures (10^−2^ mbar) and electron currents (10 mA), where secondary reactions of formed DEA products—including chemical ionisation reactions—were likely possible. Nevertheless, their spectra provides an overview of possible anionic species from His. One of the species reported in Voigt and Schmidt^29^ is [His–COOH]^−^, *m/z* 110, which is also observed in the present study. However, the corresponding ion yield shown in Figure 4B turned out to be very low. Therefore, we omit a more detailed data analysis, and we just have computationally investigated the formation of this anion in more detail. Assuming a single cleavage of the C_β_−C bond, the resulting [His–COOH]˙ radicals will have slightly negative AEA of −0.21 eV for ^τ^His and slightly positive AEA of 0.14 eV for ^π^His. The former anion will be unstable towards spontaneous emission of the excess electron (thus not observable with the present experimental set‐up). In both cases, this reaction has substantial free energy of reaction and could be observable above the thermodynamic threshold of 3.51 eV for ^τ^His and 3.06 eV for ^π^His. When two neutral products are formed, ie, CO_2_ + H˙, the thresholds lower to 3.37 and 2.92 eV, respectively. If a rearrangement reaction is considered, where the H˙ from the N position of the imidazole moiety is transferred to the C_β_ carbon, the thermodynamic threshold for the release of two neutrals CO_2_ + H˙ lowers to 0.61 eV for ^τ^His and 0.55 eV for ^π^His. Such lowering of the threshold results from the much higher AEA of 2.65 eV for the radical formed. The structure of [His–COOH]^−^ is shown in Figure 5. Interestingly, in DEA studies with other amino acids, only the complementary anion was observed, ie, the anion HCO_2_^−^.^19^, ^20^, ^64^, ^65^, ^66^ The latter anion is not observed within the detection limit of the present apparatus. This shows that the imidazole moiety, which has released the H˙ from the N position, retains preferentially the captured electron, which makes sense due to its particular high electron affinity (2.6 eV).^70^

Figure 4C shows the anion yield of even electron ion at *m/z* 81, [C_4_N_2_H_5_]^−^. The ion yield shows three low‐lying peaks at 0.05, 0.27, and 1.07 eV with the experimental threshold at ~0 eV electron energy. If such fragment ion is formed upon DEA to His, the imidazole moiety keeps the excess charge also in this reaction and the backbone of the amino acid, H_2_NCHCOOH, is lost. Simple C_α_−C_β_ bond cleavage would lead to the imidazole radical with a rather substantial AEA of 2.35 eV for ^τ^His and 1.77 eV for ^π^His. However, the reaction would still be endothermic by 1.76 and 1.20 eV, respectively, and no peak is observed above this energy experimentally. Therefore, the formation of [C_4_N_2_H_5_]^−^ may be based on molecular rearrangement with transfer of the H˙ from the N position of the imidazole ring to the C_α_ carbon (see Figure 5). This structure has an AEA of 2.47 eV, which is even higher than the imidazole radical formed by simple C_α_−C_β_ bond cleavage. Additionally, it makes the DEA reaction for both tautomers slightly exothermic by −0.31 for ^τ^His and −0.37 eV for ^π^His, which is in agreement with the experimental results. We briefly note that, analogous for positive ions, we may exclude formation of [C_4_N_2_H_5_]^−^ by single H‐loss from 4‐Methylimidazole, [C_4_N_2_H_6_], formed as thermal decomposition product from His. We determined the thresholds in these cases and obtained 1.24 and 1.22 eV for the H‐loss from N1 and N3 positions of the imidazole ring, respectively which can be expected as energetically most favourable reaction. Therefore, the first three peaks of the [C_4_N_2_H_5_]^−^ ion yield shown in Figure 4C cannot be formed due to DEA to 4‐Methylimidazole.

In the course of the present DEA studies, we additionally found ion yields at *m/z* 17 and *m/z* 16. The corresponding anions are OH^−^ and O˙^−^/NH_2_^−^, respectively. Ion yields at these two masses have also been observed in anion desorption experiments with condensed films of His, which were irradiated by low‐energy electrons.^30^ Furthermore, these anions were observed in DEA studies of other amino acids in the gas phase (see, eg, Ptasinska et al^19^).

The present ion yields at *m/z* 16 and 17 are characterised by a very rich pattern of peaks from ~0 eV electron energy up to 15 eV (see Table 3 for exact positions). The OH^−^ anion may be formed by simple C−OH bond cleavage. The corresponding neutral fragment is shown in Figure 5. The anion yield of OH^−^ is depicted in Figure 4D. Quantum chemical calculations show that the C−OH bond dissociation energy is 4.89 eV for ^τ^His and 4.74 eV for ^π^His. The calculated AEA of the ˙OH radical of 1.68 eV makes the DEA reaction endothermic by 2.75 eV for ^τ^His and 2.63 eV for ^π^His. Separation of CO from the formed neutral lowers slightly the free reaction energy to 2.44 eV for ^τ^His and 2.37 eV for ^π^His. Both values are still substantially above the experimental threshold of (0.04 ± 0.03) eV. Hence, the ion yield below about 2.4 eV (covering the resonance peaks at 0.47, 1.00, and 2.29 eV) arises from another (so far unknown) process than DEA of a single electron to His. We note that a highly abundant peak at zero eV (exceeding features at higher energies by a factor of about 10) was observed for OH^−^ in DEA to tryptophan.^20^ The origin of this signal remained unclear. In contrast, for proline and aliphatic amino acids, features in the OH^−^ ion yields close to zero eV were rather weak and lower in intensity compared with features at higher energies, and they were assigned to hot band transitions^65^ or impurities.^71^ The most prominent peak observed in the present study is located at the electron energy of 7 eV (see Figure 4D). In comparison, the ion yield of OH^−^ desorbed from an electron bombarded condensed film of His showed a peak at the electron energy^30^ of 7.7 eV noting also a shoulder of another faint peak. Therefore, we may assign these peaks observed in the gas phase and the condensed phase to the same TNI state of His, in view that anions formed with low kinetic energies—representing the low‐energy tail of a resonance—may be more strongly discriminated against in the desorption process than anions with higher kinetic energies.^72^

The most abundant anion observed in the present experiments is found at *m/z* 16, assigned to O˙^−^/NH_2_^−^. The mass resolution of the used quadrupole mass spectrometer is not sufficient to separate these isobaric anions. The measured anion yield is shown in Figure 4E, and the corresponding neutrals formed are shown in Figure 5, if formed from His. The anion yield shows 5 peaks above the experimental threshold of (3.9 ± 0.1) eV (Table 3). Utilising a double focusing mass spectrometer, it was shown in previous DEA studies to the amino acids valine,^22^ beta alanine^73^ and glycine^74^ that both isobaric ions were formed. These studies showed that NH_2_^−^ is formed with a threshold of ~4 to 5 eV dominating at electron energies of 5 to 8 eV, whereas O˙^−^ was observed dominantly in a peak close to 4 eV and in the region of 7 to 15 eV. In the valine ion yield measured previously,^22^ the O˙^−^ peak close to 4.4 eV and a second peak at 8.2 eV resembled the characteristics^75^, ^76^ of O˙`^−^ from CO_2_ and were ascribed to thermally decomposed valine. Also in the present case, CO_2_ is present in the molecular beam (see above), and therefore, the first peak at about 4.3 eV may be assigned to O˙^−^/CO_2_. The second well‐known peak^75^, ^76^ at 8.2 eV in the O˙^−^ from CO_2_ may be obscured due to stronger abundant peak at 9.2 eV. We further note that the latter peak may also arise from water impurities in the sample. This hypothesis is further supported by the presence of other peaks at about 7 and 11.7 eV which were also found for O˙^−^ from H_2_O.^77^ Therefore, one may conclude that only the peak at 5.74 eV found experimentally results from DEA to His, corresponding to formation of the NH_2_^−^ fragment ion.

Our quantum chemical calculations predict that the thermodynamic threshold for removal of O˙^−^ from C═O requires 5.56 eV for ^τ^His and 5.72 eV for ^π^His. However, removal of O˙^−^ from the −COOH site with H transfer to C (see Figure 5) lowers the threshold to 3.65 eV for both His tautomers. On the other hand, NH_2_^−^ formation by simple bond cleavage has even a lower free reaction energy of 2.61 eV for ^τ^His and 2.31 eV for ^π^His (see Figure 5). The lower threshold for NH_2_^−^ formation can be explained by the weaker C_β_−N bond, 3.78 eV for ^τ^His and 3.44 eV for ^π^His, in comparison with the C═O bond dissociation energy of 7.34 and 7.50 eV, respectively.

## 5 CONCLUSIONS

In the present study, we investigated electron ionisation of histidine close to threshold of the different fragments and dissociative electron attachment in the electron energy range from about 0 up to 18 eV. The experimental data were supported by quantum chemical calculations, which derived free energies of reactions and allowed the identification of possible neutral and charged products produced. Generally, the quantum chemical calculations are in good agreement with the experimental data within the estimated uncertainties. The experimentally determined ionisation energy of His agrees with the calculated vertical ionisation energy of the ^π^His tautomer. In addition to the parent ion, we investigated the ionisation threshold behaviour of four fragment ions formed upon electron ionisation of His. The remarkable situation for [His–COOH]^+^ occurs that its appearance energy in case of the τ tautomer is very close to the ionisation energy of the His molecule. The calculated value indicates that formation of [His–COOH]^+^ is possible even below the ionisation energy of the parent; however, the accuracy of the corresponding experimental values does not allow a direct conclusion. Such unusual order of ionisation and appearance energies is very scarce in electron ionisation and was previously reported for CHF_2_Cl.^78^

In the case of negative ion formation, five fragment anions were obtained in the course of the present studies. Like for all other studied aromatic (as well as aliphatic) amino acids so far, no molecular anion was observed within the detection limit of the apparatus, which indicates that the temporary negative ion undergoes fast spontaneous emission of the excess electron or dissociation into stable fragment anions and neutral(s). The ion yield of the dehydrogenated parent anion does not show the characteristic shape of a steep onset close to 1.2 eV obtained for other amino acids like glycine and formic acid. The absence of this onset may be explained by the lowered threshold compared to glycine^79^ as well as the possibility of H‐loss from the imidazole moiety. For other dissociation channels, we investigated the possibility of structural rearrangement, which finally supports the measured threshold in the ion yields of [His–COOH]^−^ and [C_4_N_2_H_5_]^−^.

The previous studies by Wilson et al^31^ suggest that a large fraction of the His sample decomposes upon thermal heating (a standard technique for sublimation of nonvolatile samples). The present study does not confirm this previous conclusion. By comparison with the mass spectra of possible decomposition products and calculated thresholds for positive and negative ion formation, it is possible to assign the obtained ion yields to reactions of intact histidine in most cases. This result is in agreement with the previous studies of the His mass spectrum based on the laser‐induced acoustic desorption technique,^53^, ^54^ which is a gentle method to transfer neutral molecule into the gas phase. The only volatile thermal decomposition product of His detected in the present mass spectrum is carbon dioxide, which was straightforward to identify in the electron energy scans due its well‐known ionisation energy and resonance energies.

## CONFLICT OF INTEREST

The authors declare that they have no conflict of interest.

## Supporting information

Data S1. Supporting Information
