# Supplementary material for: Positive and negative ions of the amino acid histidine formed in low‐energy electron collisions
Source: J Mass Spectrom. 2019 Nov 6;54(10):802–16. doi: 10.1002/jms.4427 (PMC6916310; doi:10.1002/jms.4427)
Supplement: Supplementary file 1 — Data S1. Supporting Information [file JMS-54-802-s001.pdf]

Positive and negative ions of the amino acid histidine formed in low-energy electron collisions

Journal of Mass Spectrometry

Rebecca Meißner,[ab] Linda Feketeová,[ac] Andreas Bayer,[a] Johannes Postler,[a] Paulo Limão-Vieira[b] and Stephan Denifl[a]

[a]Institut für Ionenphysik und Angewandte Physik and Center for Molecular Biosciences Innsbruck (CMBI), Universität Innsbruck, Technikerstraße 25, 6020 Innsbruck, Austria

[b]Atomic and Molecular Collisions Laboratory, CEFITEC, Department of Physics, Universidade NOVA de Lisboa, 2829-516 Caparica, Portugal

[c]Institut de Physique Nucléaire de Lyon; CNRS/IN2P3, UMR5822, Université de Lyon, Université Claude Bernard Lyon 1, 43 Bd du 11 novembre 1918, 69622 Villeurbanne, France

Corresponding authors:
Rebecca Meißner,[a] rebecca.meissner@uibk.ac.at, +43 (0)512-507-52771;
Stephan Denifl,[a] stephan.denifl@uibk.ac.at;
Linda Feketeová,[b] l.feketeova@ipnl.in2p3.fr

A) Ionization of Histidine $C_6H_9N_3O_2$

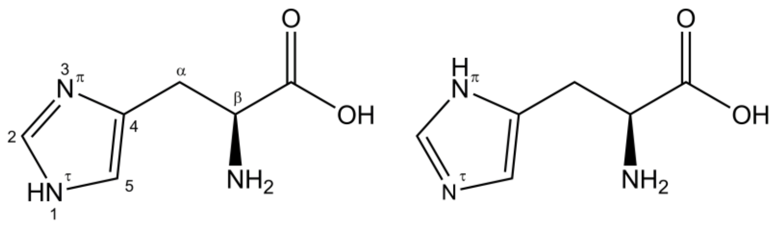

$^{\tau}$His (0.0eV)

$^{\pi}$His (0.08eV)

$^{\tau}$His	
M062x/aug-cc-pVTZ:	DSD-PBEP86:
AIE = 8.30	8.33
VIE = 8.86	8.88

$^{\pi}$His	
M062x/aug-cc-pVTZ:	DSD-PBEP86:
AIE = 8.13	8.11
VIE = 8.45	8.44

AIE= Adiabatic ionization energy; VIE= Vertical Ionization energy
All values are in eV.

[His – COOH]$^+$
m/z 110

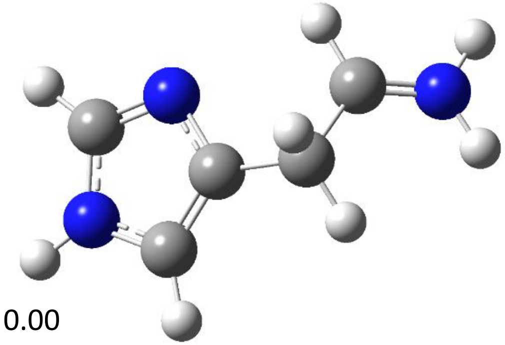

Δ = 0.00

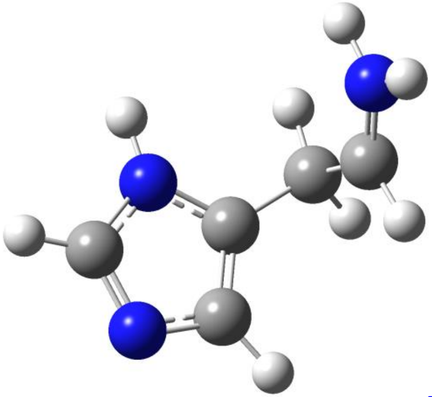

AIE = 5.92 eV

8.67 eV	(9.25 eV)	+ CO_2H	9.12 eV	(9.67 eV)
**8.53 eV**	(9.33 eV)	+ CO_2 + H	**8.98 eV**	(9.74 eV)

AIE = 4.96 eV

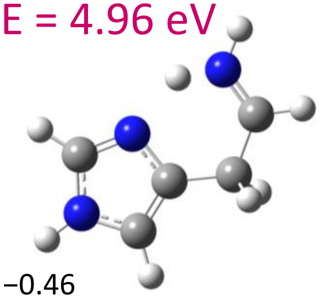

Δ = −0.46

8.24 eV (8.82 eV) + CO_2H

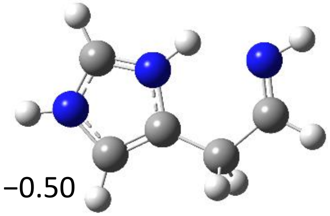

Δ = −0.50

8.21 eV (8.76 eV) + CO_2H

The values are reaction free energies in eV for the formation of the fragment ion and neutrals. Values in green correspond to ΔE_{0K} for the corresponding process. The relative stability of the fragment ions on the left is also shown.

$[C_4N_2H_6]^{\bullet+}$
m/z 82

$^{\tau}$His (0.0eV)

$^{\pi}$His (0.08eV)

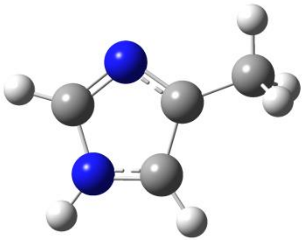

AIE = 8.39 eV

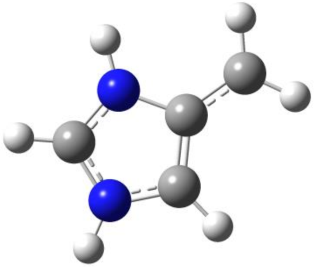

AIE = 5.56 eV

Ion

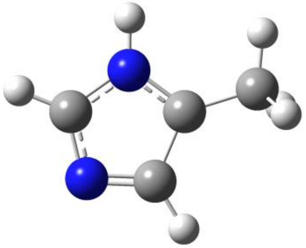

AIE = 8.35 eV

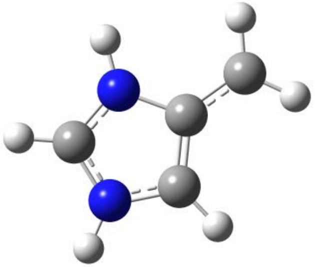

AIE = 5.56 eV

+ neutrals:

+ 73 ($H_2NCCOOH$)	10.20	9.53
+ 73 (HNCHCOOH)	**8.85** (9.44)	8.21
+ CO_2H + H_2NC	13.06	12.41
+ CO_2 + H_2NCH	9.75	9.10
**+ CO_2 + H_2 + HCN**	**8.62** (9.75)	7.97 (9.07)

+ 73 ($H_2NCCOOH$)	10.07	9.46
+ 73 (HNCHCOOH)	**8.74** (9.34)	8.14
+ CO_2H + H_2NC	12.95	12.35
+ CO_2 + H_2NCH	9.64	9.03
**+ CO_2 + H_2 + HCN**	**8.51** (9.64)	7.90 (8.99)

The values are reaction free energies in eV for the formation of the fragment ion and neutrals. Values in green correspond to ΔE_{0K} (eV) for the corresponding process.

$[C_4N_2H_5]^+$
m/z 81

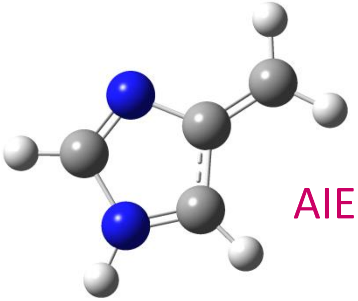

AIE = 6.92 eV

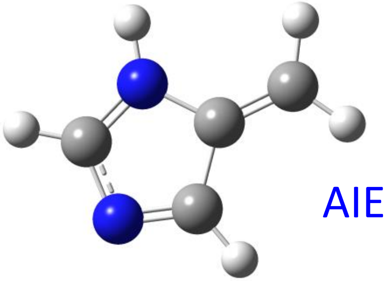

AIE = 7.03 eV

+ neutrals:

+ 74	8.99 (10.02)	+ 74	8.93 (9.95)
**+ CO_2 + H_2 + H_2CN**	**9.73** (11.01;9.09)	**+ CO_2 + H_2 + H_2CN**	**9.67** (10.94; 9.04)
+ CO_2 + H_2 + H + HCN	10.79	+ CO_2 + H_2 + H + HCN	10.73
+ CO_2H + H_2 + HCN	10.93	CO_2H + H_2 + HCN	10.86

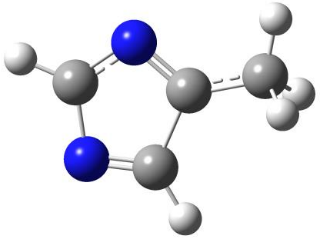

+ CO_2 + H_2 + H + HCN 12.97 // 12.90

+ CO_2H + H_2 + HCN 13.11 // 13.04

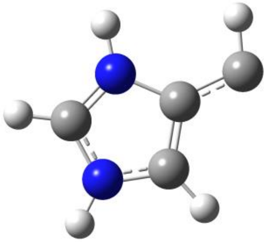

+ CO_2H + H_2 + HCN 12.99 // 12.93

The values are reaction free energies in eV for the formation of the fragment ion and neutrals. Values in green correspond to ΔE_{0K} (eV) for the corresponding process and values in orange are reaction free energies at 433K.

$[NH_3CHCH_2]^+$
m/z 44

$(NH_2{=}CH{-}CH_3^+)$ + 66 + CO_2H	14.27	$(NH_2{=}CH{-}CH_3^+)$ + 66 + CO_2H	13.03
$(NH_2{=}CH{-}CH_3^+)$ + 66 + CO_2 + H	14.13	$(NH_2{=}CH{-}CH_3^+)$ + 66 + CO_2 + H	12.88
$(NH_2{=}CH{-}CH_3^+)$ + 66 (chain) + CO_2 + H	15.15	$(NH_2{=}CH{-}CH_3^+)$ + 66 (chain) + CO_2 + H	12.26
$(NH_2{=}CH{-}CH_3^+)$ + 67 + CO_2	8.97	$(NH_2{=}CH{-}CH_3^+)$ + 67 + CO_2	9.05

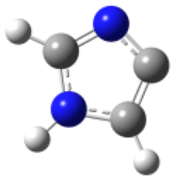
67

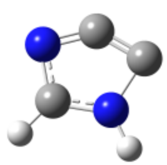
66

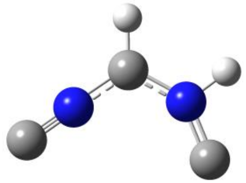
66 (chain)

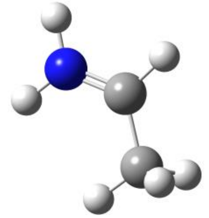
$(NH_2{=}CH{-}CH_3^+)$

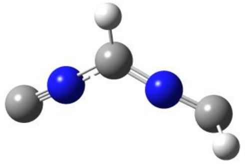
66 (chain)

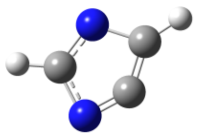
66

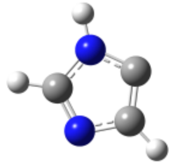
67

$(NH{=}CH{-}NH_2^+)$ + CO_2 + pyrrole		8.73	$(NH{=}CH{-}NH_2^+)$ + CO_2 + pyrrole		8.67
**$(NH{=}CH{-}NH_2^+)$ + CO_2 + chain**	(11.45)	**10.41**	**$(NH{=}CH{-}NH_2^+)$ + CO_2 + chain**	(11.37)	**10.34**

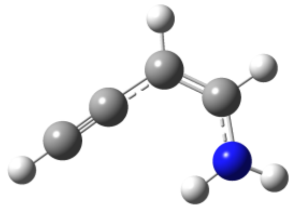
chain

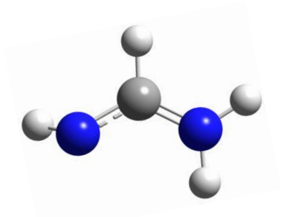
$(NH{=}CH{-}NH_2^+)$

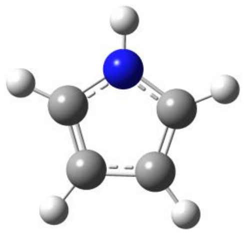
pyrrole

The values are reaction free energies in eV for the formation of the fragment ion and neutrals. Values in green correspond to ΔE_{0K} (eV) for the corresponding process.

$[NH_3CHCH_2]^+$
m/z 44

$(NH_3\text{-}CH\text{-}CH_2^+) + 67 + CO_2$	9.63	$(NH_3\text{-}CH\text{-}CH_2^+) + 67 + CO_2$	9.71
$(NH_3\text{-}CH\text{-}CH_2^+) + 66 + H + CO_2$	14.79	$(NH_3\text{-}CH\text{-}CH_2^+) + 66 + H + CO_2$	13.55
$(NH_3\text{-}CH\text{-}CH_2^+) + 66 + CO_2H$	14.93	$(NH_3\text{-}CH\text{-}CH_2^+) + 66 + CO_2\,H$	13.69

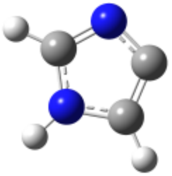
67

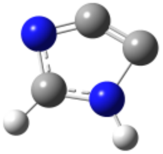
66

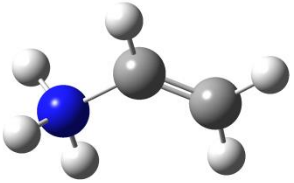
$(NH_3\text{-}CH\text{-}CH_2^+)$

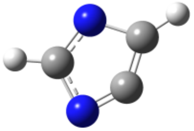
66

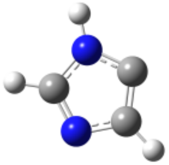
67

The values are reaction free energies in eV for the formation of the fragment ion and neutrals.

$CO_2^{\bullet +}$
m/z 44

AIE (CO_2) = 13.94 eV (calculated)

(CO_2^+) + 82 + H_2 + HCN	14.14	(CO_2^+) + 82 + H_2 + HCN	14.10
(CO_2^+) + 111	13.45	(CO_2^+) + 111	13.33
(CO_2^+) + 110 + 1	17.02	(CO_2^+) + 110 + 1	16.94

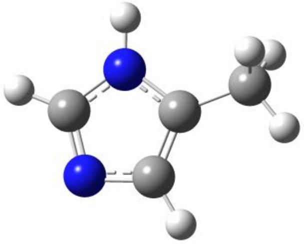

82

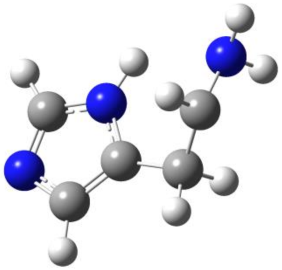

110

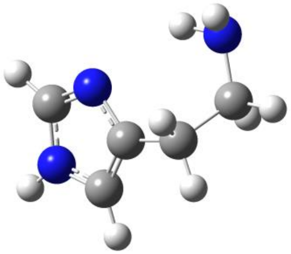

111

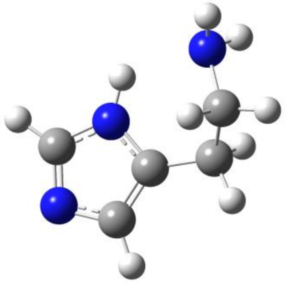

111

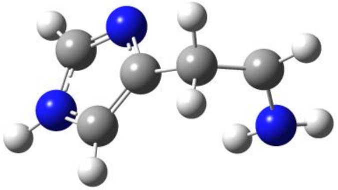

110

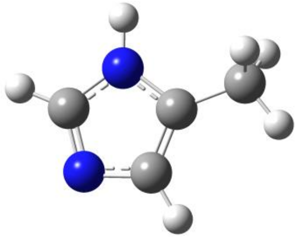

82

The values are reaction free energies in eV for the formation of the fragment ion and neutrals.

B) Electron attachment to Histidine $C_6H_9N_3O_2$

$^{\tau}$His

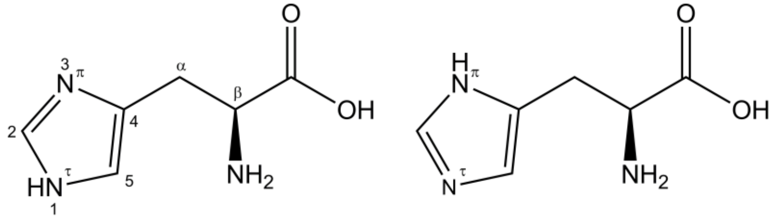

$^{\pi}$His

τ tautomer **1a**

π tautomer **1b**

L-Histidine **1**

$^{\tau}$His (0.0eV)

$^{\pi}$His (0.08eV)

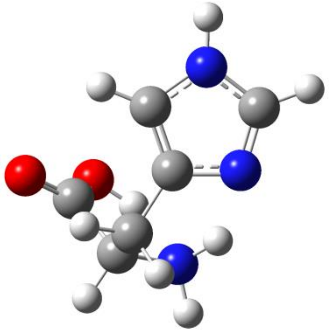

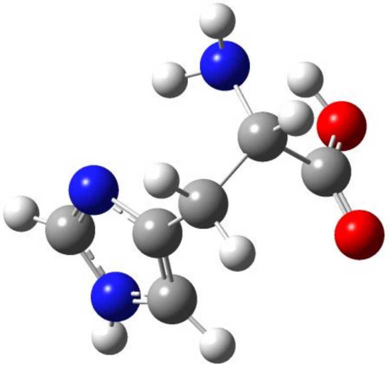

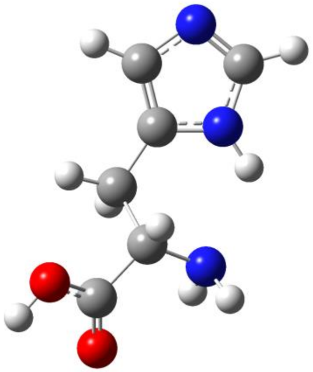

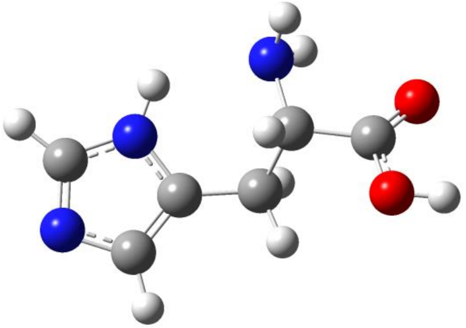

[His – COOH]$^-$
m/z 110

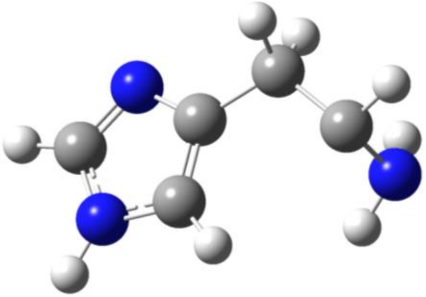

AEA = −0.21 eV

**+ CO_2H**	**3.51**	(4.07)
**+ CO_2 + H**	**3.37**	(4.15)

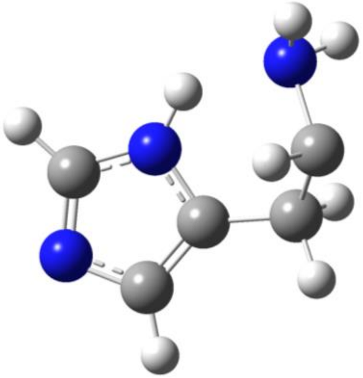

AEA = 0.14 eV

**+ CO_2H**	**3.06**	(3.61)
**+ CO_2 + H**	**2.92**	(3.69)

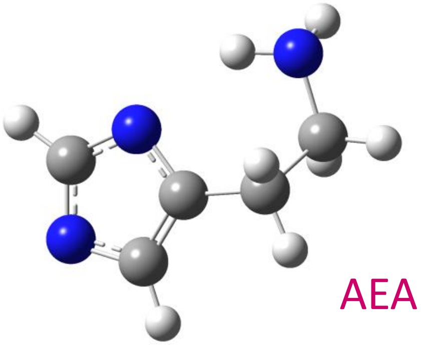

AEA = 2.65 eV

**+ CO_2H**	**0.76**	(1.31)
**+ CO_2 + H**	**0.61**	(1.39)

same energy ⟷

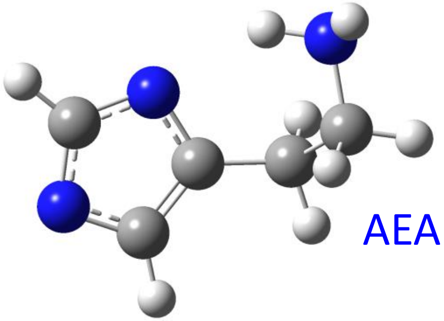

AEA = 2.65 eV

**+ CO_2H**	**0.69**	(1.23)
**+ CO_2 + H**	**0.55**	(1.31)

AEA = Adiabatic electron affinity

The values are reaction free energies in eV for the formation of the fragment ion and neutrals. Values in green correspond to ΔE_{0K} (eV) for the corresponding process.

$[C_4N_2H_5]^-$
m/z 81

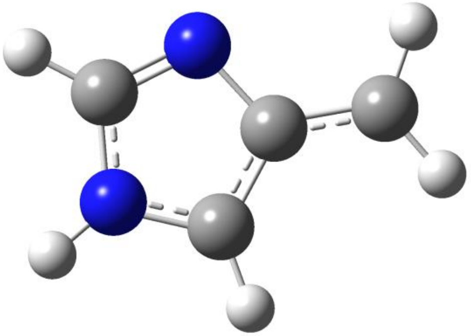

AEA = 2.35 eV

+

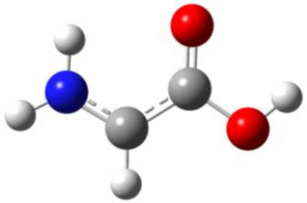

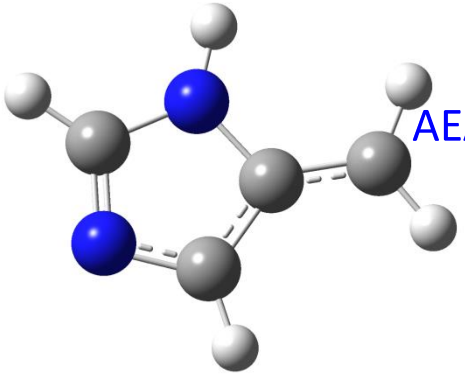

AEA = 1.77 eV

1.76 (2.35)

1.20 (1.77)

+ H_2NCH + CO_2H	4.82	+ H_2NCH + CO_2H	4.26
+ H_2NCH + CO_2 + H	4.68	+ H_2NCH + CO_2 + H	4.12
**+ H_2NCH_2 + CO_2**	**1.84**	**+ H_2NCH_2 + CO_2**	**1.27**

$[C_4N_2H_5]^-$
m/z = 81
-0.31 (0.29) **AEA = 2.47**

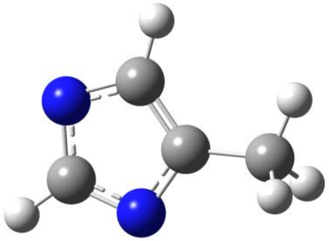

-0.37 (0.21)

The values are reaction free energies in eV for the formation of the fragment ion and neutrals. Values in green correspond to ΔE_{0K} (eV) for the corresponding process.

O^-
m/z 16

5.56 (5.93)

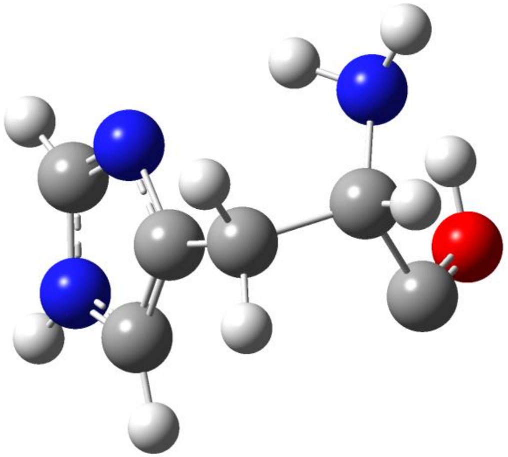

[His – O]

5.72 (6.09)

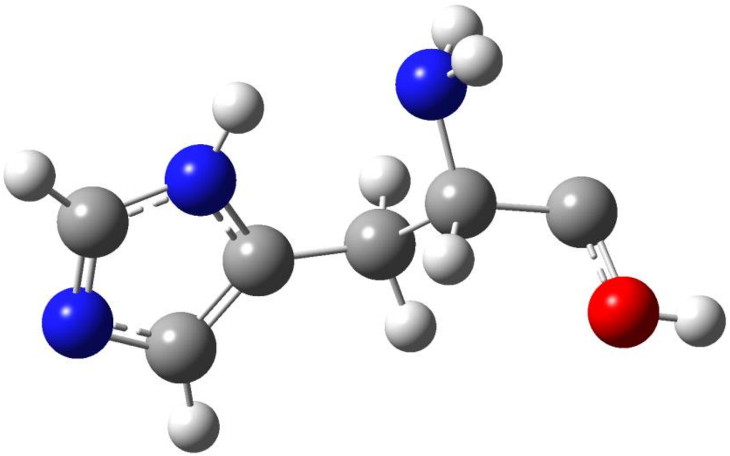

The values are reaction free energies in eV for the formation of the fragment ion and neutrals. Values in green correspond to ΔE_{0K} (eV) for the corresponding process.
